# Posterior reversible encephalopathy syndrome in the pediatric population: a pictorial essay

**DOI:** 10.1590/0100-3984.2021.0148

**Published:** 2022

**Authors:** Filipa Proença, Manuel Alberto Correia, Graça Nunes, Lia Lucas Neto

**Affiliations:** 1 Serviço de Imagiologia Neurológica, Centro Hospital Universitário Lisboa Norte – Hospital Santa Maria, Lisboa, Portugal.; 2 Instituto de Anatomia, Faculdade de Medicina da Universidade de Lisboa, Lisboa, Portugal.

**Keywords:** Posterior leukoencephalopathy syndrome/diagnostic imaging, Neuroimaging, Magnetic resonance imaging, Child, Adolescent, Síndrome da leucoencefalopatia posterior/diagnóstico por imagem, Neuroimagem, Ressonância magnética, Criança, Adolescente

## Abstract

Posterior reversible encephalopathy syndrome (PRES) is a rare disease attributed to an increase in blood pressure that exceeds the autoregulatory capabilities of the cerebral vasculature, resulting in brain edema. Although PRES primarily affects adults, the pediatric population is also at risk. Radiologists must be aware of that risk because the imaging features on brain MRI are often atypical, especially in pediatric patients. Over a 6-year period, nine pediatric patients were diagnosed with PRES at our institution. Here, those patients are evaluated retrospectively regarding demographic characteristics, clinical profiles, imaging aspects, and outcomes. In this pictorial essay, we review the typical and atypical imaging findings of PRES in pediatric patients, demonstrating that it should be considered in patients with a clinical profile suggestive of the diagnosis, given that prompt, effective treatment is important for full recovery, thus avoiding major morbidity and mortality in such patients.

## Abstract (pt)

A síndrome de encefalopatia posterior reversível (PRES) é uma doença rara relacionada com uma falha no mecanismo autorregulatório vascular, resultando em edema cerebral. Apesar de primariamente descrita em adultos, a população pediátrica encontra-se em igual risco de desenvolver esta condição e o radiologista deve estar atento porque frequentemente a ressonância magnética apresenta características de imagem atípicas. Em um período de seis anos, nove pacientes pediátricos com o diagnóstico de PRES foram avaliados, retrospectivamente, no que diz respeito a sua demografia, antecedentes médicos, características de imagem e prognóstico. Neste ensaio analisamos características de imagem típicas e atípicas de pacientes pediátricos com o diagnóstico de PRES, mostrando que este diagnóstico deve ser considerado quando está presente um quadro clínico adequado, uma vez que o tratamento rápido e eficaz é importante para a recuperação completa, evitando morbimortalidade maior neste grupo etário.

## INTRODUCTION

Posterior reversible encephalopathy syndrome (PRES), first described in adults in 1996^(1)^, is a rare, controversial syndrome whose pathophysiology is not fully understood. One of the main mechanisms of PRES is the failure of cerebral vascular regulatory mechanisms in the context of sudden blood pressure changes. To date, three etiological hypotheses have been proposed^(2)^: vasoconstriction leading to infarction; failure of vascular autoregulation, resulting in vasogenic edema; and endothelial damage with disruption of the blood–brain barrier, leading to transudation of fluid and proteins. Although PRES has been reported to be more common in adults, the pediatric population is also at risk for the syndrome. However, data in the literature regarding PRES in the pediatric population are scarce and less robust, most being from retrospective single-center studies that focused on a specific subset of patients^(3,4)^. The exact overall incidence of PRES remains unknown^(5)^. However, recent data indicate that the incidence is 0.04% among hospitalized children^(3)^.

A wide spectrum of risk factors for and triggers of PRES in the pediatric population has been described, including hypertensive encephalopathy, renal failure, immunosuppressive or cytotoxic drug use, oncologic diseases, thrombocytopenia, and sepsis. The brains of children and adolescents differ from those of adults in terms of vulnerability, hemodynamic responses, and vascular regulation. Therefore, the course of the disease differs as well^(4)^. Because studies of pediatric PRES have appeared relatively recently in comparison with those addressing it in adults, pediatricians seem to have little experience with this syndrome^(4)^. Clinically, PRES is characterized by various neurological signs and symptoms, such as headaches, visual disturbance, seizures, and even altered mental status, although it can be overlooked by clinicians^(4)^.

Neuroimaging evaluation plays an important role in the definite diagnosis of PRES, and MRI, which can show even small lesions, is considered the gold standard^(6)^. Classical brain MRI findings in PRES include reversible bilateral, symmetric white matter lesions, cortical signal changes in the parietal and occipital regions on T2-weighted fluid-attenuated inversion recovery (FLAIR) sequences, together with atypical imaging findings such as asymmetric or unilateral lesions, as well as posterior fossa lesions, contrast-enhancing lesions, and lesions that are hemorrhagic or show restricted diffusion on diffusion-weighted imaging (DWI), contributing to the controversy surrounding the syndrome. Such alterations are usually reversible, and a DWI study might help in differentiating between reversible vasogenic edema and cytotoxic edema/ischemic lesions. According to the literature, these atypical findings are more common among pediatric patients, although they do not always correlate with the severity of symptoms. That can create a diagnostic dilemma, resulting in delayed diagnosis and treatment, which can lead to poor outcomes^(7)^.

In this pictorial essay, we review typical and atypical imaging findings of PRES in children and adolescents treated in the intensive care unit of our hospital, demonstrating that it should be considered in patients with a clinical profile suggestive of the diagnosis, given that prompt, effective treatment is important for full recovery, thus avoiding major morbidity and mortality in this population. The main demographic, clinical, and imaging data are detailed in Table 1.

**Table 1:** Demographic characteristics, clinical profiles, outcomes, and imaging findings of pediatric patients diagnosed with PRES.

Patient	Sex	Age (years)	Medical history	CTX	Symptoms	MV	BP* (mmHg)	LOS (days)	Outcome at 90 days	Location of lesions on brain MRI	Other MRI aspects	Recovery time
1	F	13	T1DM, anemia, and AKI		Altered mental status, HBP, seizures, and headache	Y	166/99†	3	Gait abnormality	Parietal lobe (bilaterally); right occipital lobe; and frontal lobe	Blooming (hemorrhage)	11 days
2	F	4	Burkitt’s lymphoma	COP + rituximab + methotrexate	HBP and seizures	N	157/96†	2	LTFU	Parietal and frontal lobes (bilaterally)	—	LTFU
3	F	8	ALL	DFCI 05-001	Altered mental status and HBP	N	140/60†	2	Symptom reversal	Parietal and occipital lobes (bilaterally); and left frontal lobe	—	LTFU
4	M	18	Stage IV seminoma	TIP	Seizures	N	120/86	16	Death	Cerebellar hemispheres; right parieto-occipital region; parietal and frontal lobes (bilaterally); basal ganglia (bilaterally); and left corona radiata	—	4 days
5	M	7	Nephrotic syndrome (AKI), cerebral venous thrombosis	—	Altered mental status and HBP	N	136/84†	2	Dysfunctional behavior and headaches	Cerebellar hemispheres; right frontal-temporal-occipital-parietal region; and left parietal and frontal lobes	—	4 months
6	M	14	Supraventricular tachycardia (cardiogenic shock and AKI)	—	HBP and seizures	Y	104/65	20	Symptom reversal	Frontal-parietal-occipital region (bilaterally); and right cerebellar hemisphere	Blooming (hemorrhage)	5 months
7	M	10	FIRES and AKI	—	HBP and status epilepticus	Y	133/103†	83	Epilepsy	Parietal and occipital lobes (bilaterally); and left frontal lobe	—	3 months
8	F	11	ALL and sepsis	DFCI 05-001	Status epilepticus	Y	118/76	12	Death	Left parietal and occipital lobes	—	5 days
9	F	8	Nephrotic syndrome and myocardiopathy	—	Seizures, headache, visual disturbance, and vomiting	N	159/105†	7	Symptom reversal	Frontal lobe (bilaterally); left parietal-occipital region; and cerebellar hemispheres	Restricted diffusion	1 month

F, female; M, male; T1DM, type 1 diabetes mellitus; AKI, acute kidney injury; ALL Consortium protocol (vincristine + dexamethasone + 6-mercaptopurine + doxorubicin + methotrexate + asparaginase + cytarabine + hydrocortisone); FIRES, febrile infection-related epilepsy syndrome; CTX, chemotherapy; COP, cyclophosphamide + vincristine + prednisone; DFCI 05-001, Dana-Farber Cancer Institute ALL Consortium protocol (vincristine + dexamethasone + 6-mercaptopurine + doxorubicin + methotrexate + asparaginase + cytarabine + hydrocortisone); TIP, paclitaxel + ifosfamide + cisplatin; HBP, high blood pressure; MV, mechanical ventilation; Y, yes; N, no; BP, blood pressure; LOS, length of stay; LTFU, lost to follow-up.

* Mean baseline values.

† HBP, according to age and gender percentile charts.

Over a 6-year period, nine pediatric patients were diagnosed with PRES. Five (60%) of those patients were female. The mean age was 10.33 years, the majority of the patients being teenagers. The primary underlying diseases were type 1 diabetes mellitus, anemia, Burkitt’s lymphoma, acute lymphoblastic leukemia, seminoma, nephrotic syndrome, and supraventricular tachycardia. One patient had no relevant personal medical history. Four patients had oncologic disease and were under chemotherapy. The clinical presentations of PRES included seizures, status epilepticus, reduced level of consciousness, headache, and visual disturbance. In addition, most of the patients developed high blood pressure. Three patients were lost to follow-up: two died, and one was transferred to another facility. At 90 days of follow-up, three of the six remaining patients were asymptomatic and the other three developed gait abnormality, headaches plus dysfunctional behavior, and epilepsy, respectively. In most cases, brain MRI revealed at least one atypical imaging feature. In the T2-weighted FLAIR sequence, areas of signal hyperintensity were identified in the biparietal region, occipital region, frontal lobe, temporal lobe, basal ganglia, and cerebellum. In one case, DWI showed slightly restricted diffusion in those same areas. In two other cases, hemorrhages were identified in T2*- weighted sequences. By 90 days of follow-up, the imaging abnormalities had reversed in most of the patients.

## IMAGING CHARACTERISTICS OF PRES IN THE PEDIATRIC POPULATION

All brain MRIs were performed in 1.5-T scanner, with the following sequences: sagittal T1-weighted sequences with multiplanar three-dimensional reconstruction; axial proton density, T2-weighted sequences; T2-weighted FLAIR sequences; T2*-weighted sequences; DWI with apparent diffusion coefficient mapping; and coronal T2- weighted sequences. The majority of the patients had impaired renal function and therefore underwent unenhanced MRI. The images were analyzed by neuroradiologists and discussed with the pediatric team when appropriate. The MRI examinations were repeated if warranted by the clinical profile of the patient and at the discretion of the clinician.

### Typical imaging findings

**Figure 1:**
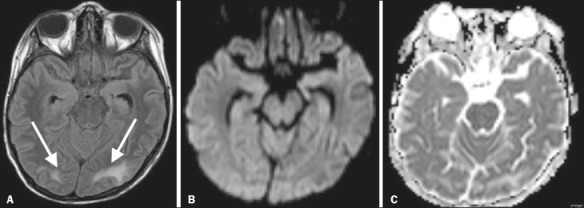
Brain MRI. Axial T2-weighted FLAIR sequence (A) showing lesions in typical parieto-occipital regions (arrows) without restricted diffusion on DWI with apparent diffusion coefficient mapping (B and C).

**Figure 2:**
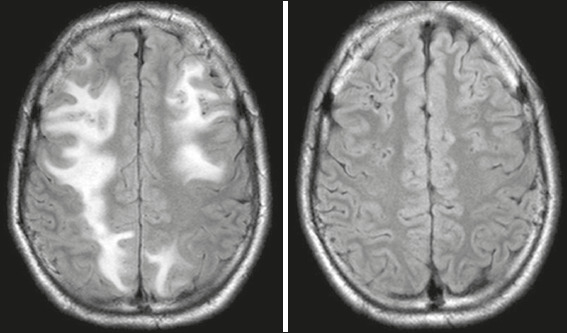
Brain MRI. T2-weighted FLAIR sequence showing the reversibility of lesions.

Although it has been reported that the lesions caused by PRES are asymmetric in 28% of cases^(8)^, the typical imaging aspects are focal regions of symmetric cortical and subcortical white matter lesions with signals that are hypointense on T1-weighted sequences and hyperintense on T2-weighted FLAIR sequences, likely representing vasogenic edema, in both parieto-occipital regions, without restricted diffusion or hemorrhage^(6,8)^, as shown in Figure 1. In the classic definition of PRES, the spontaneous disappearance of lesions is considered normal (Figure 2).

### Atypical imaging findings

#### Atypical regions and asymmetric lesions

**Figure 3:**
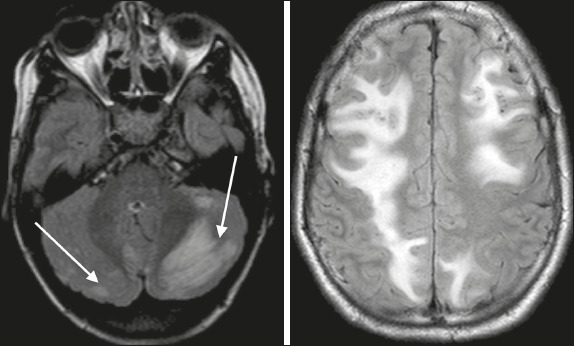
Brain MRI. Axial T2-weighted FLAIR sequence showing lesions in atypical regions: the cerebellum (arrows), frontal lobe, and parietal lobe.

Lesions attributed to PRES are not always posterior or symmetric, and atypical imaging could represent an early finding of the more classic radiological and clinical scenario^(8)^. Although the exact frequency of atypical imaging findings in pediatric PRES is unknown, it has been reported to be as high as 50% in the infratentorial region and 25–30% in the deep gray matter^(7)^. Other authors have reported that atypical MRI lesions occur in 61–82% of pediatric patients with PRES, compared with 10–58% of adults with the syndrome^(4)^. The regions most often affected, in descending order, are the parietal lobes, occipital lobes, frontal lobes, inferior temporo-occipital junction, and cerebellum; that is due to better sympathetic innervation and the consequent improvement in autoregulation of the anterior circulation^(8)^. On T2-weighted FLAIR sequences (Figure 3), lesions with hyperintense signals can be seen in atypical locations such as the frontal lobe, temporal lobe, deep gray matter, posterior fossa, brainstem, and cerebellum^(6)^. The lesions may also be asymmetric, with gadolinium enhancement, hemorrhagic changes, or restricted diffusion^(4)^. Some of these uncharacteristic imaging findings, especially extensive vasogenic edema, hemorrhage, and restricted diffusion, have been linked to poorer clinical outcomes^(7)^.

#### Restricted diffusion

**Figure 4:**
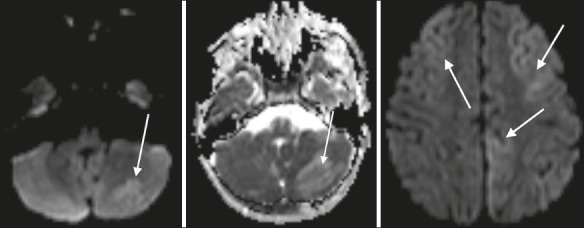
Brain MRI. Axial DWI with apparent diffusion coefficient mapping showing lesions with restricted diffusion (arrows).

Restricted diffusion (Figure 4) is considered atypical in PRES. Saad et al.^(7)^ and Chen^(4)^ reported that restricted diffusion occurs in 17–22% and 15–42% of pediatric patients with PRES, respectively, compared with 15–30% of adults with the syndrome^(4)^.

Several theories exist to explain why there are regions of restricted diffusion in some cases of PRES. The most popular theory is that hyperperfusion causes a severe mass effect from vasogenic edema with compression of the local microcirculation, resulting in acute ischemia and cytotoxic edema^(4,9)^. Chen et al.^(10)^ also demonstrated that patients who present cytotoxic edema on brain MRI are more likely to have worse outcomes and neurologic sequela. Therefore, early detection of that alteration is critical to prevent vasogenic edema from progressing to irreversibility^(9)^.

#### Hemorrhage

**Figure 5:**
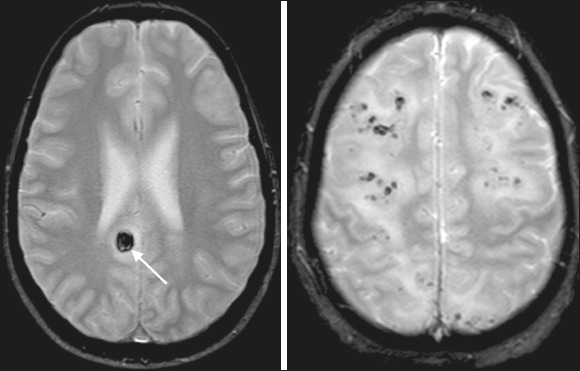
Brain MRI. Axial T2*-weighted sequence showing lesions containing hemorrhagic material (arrow).

The presence of hemorrhage on T2*-weighted imaging or (the more sensitive) susceptibility-weighted imaging^(4)^ is also considered atypical in PRES (Figure 5). In PRES, hemorrhage development may be secondary to the rupture of pial vessels in the setting of severe hypertension or reperfusion injury in the setting of vasoconstriction^(7)^. Hemorrhage reportedly occurs in 5–30% of cases, and the possible anatomical locations for its occurrence, with a similar incidence, are the brain parenchyma (focal hematoma), gyri (petechial hemorrhages), and the subarachnoid space^(8)^.

In PRES, a higher rate of microhemorrhage is associated with vasculopathy^(7,8,10)^. Patients who present with hemorrhage on brain MRI could have worse outcomes and sequelae. Although the clinical relevance of microhemorrhage is unknown, more extensive parenchymal bleeding may develop into sequelae that worsen the prognosis^(4)^. Hemorrhage might also be more common in transplant recipients, due to the underlying coagulopathy, or in patients receiving anticoagulation therapy, who constitute a major proportion of patients with PRES^(7)^.

#### Enhancing lesions

**Figure 6:**
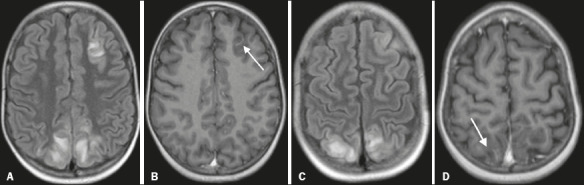
Brain MRI. Axial T2-weighted FLAIR sequences (A and C) and gadolinium contrast-enhanced T1-weighted sequences (B and D) showing enhancement in sulcal and juxtacortical lesions (arrows).

Progressive disorder of the mechanisms of cerebrovascular regulation may cause damage to the blood–brain barrier. In such cases, marked contrast enhancement can be seen on T1-weighted imaging after gadolinium administration^(8)^, as depicted in Figure 6. The reported rates of such enhancement are approximately 37–43%^(7)^. However, the presence or pattern of enhancement has not been found to show a significant association with patient outcomes^(7)^.

#### Irreversible lesions

**Figure 7:**
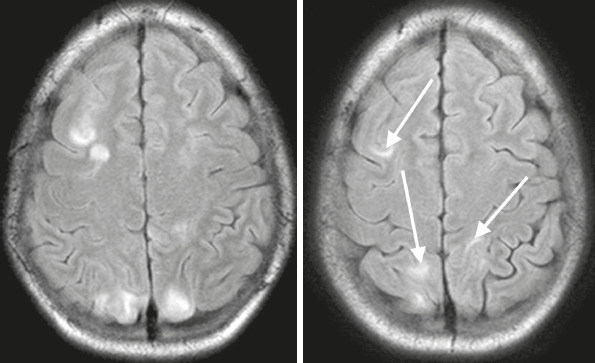
Follow-up brain MRI. Axial T2-weighted FLAIR sequence showing lesions that did not revert (arrows).

If the diagnosis is made early and treatment is applied promptly, PRES is potentially reversible, although it can also be irreversible (Figure 7). The rate of irreversible neurologic damage related to PRES has been reported to be as high as 12%. There have been reports of recurrence^(5)^ and permanent neurological sequelae, with a mortality rate of 16%^(10)^.

## CONCLUSION

Although PRES is a rare diagnosis in pediatric patients, it should not be overlooked when pediatric patients present with a clinical profile suggestive of the diagnosis. In most cases, the syndrome is reversible and has a mild course, especially if diagnosed early and treated promptly with supportive care. Imaging is fundamental for the initial diagnosis of PRES, particularly in pediatric patients, in whom atypical lesions might be more common. The fact that PRES may have an atypical presentation should be borne in mind for timely recognition and treatment of the condition. Consequently, there is a need for prospective, long-term follow-up studies to elucidate which typical or atypical imaging characteristics indicate reversibility and correlate with long-term outcomes. Such studies are especially needed in the pediatric population, in whom this syndrome may have a more severe or even life-threatening course, with sequelae and possible irreversible damage.
